# Development and Validation of a Prognostic Model to Predict the Risk of In-hospital Death in Patients With Acute Kidney Injury Undergoing Continuous Renal Replacement Therapy After Acute Type a Aortic Dissection

**DOI:** 10.3389/fcvm.2022.891038

**Published:** 2022-05-02

**Authors:** Rui Jiao, Maomao Liu, Xuran Lu, Junming Zhu, Lizhong Sun, Nan Liu

**Affiliations:** ^1^Beijing Institute of Heart, Lung and Blood Vessel Diseases, Beijing Anzhen Hospital, Capital Medical University, Beijing, China; ^2^Center for Cardiac Intensive Care, Beijing Anzhen Hospital, Capital Medical University, Beijing, China; ^3^Beijing Aortic Disease Center, Beijing Anzhen Hospital, Capital Medical University, Beijing, China

**Keywords:** acute kidney injury, acute type A aortic dissection, continuous renal replacement, in-hospital death, nomogram

## Abstract

**Background:**

This study aimed to construct a model to predict the risk of in-hospital death in patients with acute renal injury (AKI) receiving continuous renal replacement therapy (CRRT) after acute type A aortic dissection (ATAAD) surgery.

**Methods:**

We reviewed the data of patients with AKI undergoing CRRT after ATAAD surgery. The patients were divided into survival and nonsurvival groups based on their vital status at hospital discharge. The data were analyzed using univariate and multivariate logistic regression analyses. Establish a risk prediction model using a nomogram and its discriminative ability was validated using C statistic and the receiver operating characteristic (ROC) curve. Its calibration ability was tested using a calibration curve, 10-fold cross-validation and Hosmer–Lemeshow test.

**Results:**

Among 175 patients, in-hospital death occurred in 61 (34.9%) patients. The following variables were incorporated in predicting in-hospital death: age > 65 years, lactic acid 12 h after CRRT, liver dysfunction, and permanent neurological dysfunction. The risk model revealed good discrimination (C statistic = 0.868, 95% CI: 0.806–0.930; a bootstrap-corrected C statistic of 0.859, the area under the ROC = 0.868). The calibration curve showed good consistency between predicted and actual probabilities (*via* 1,000 bootstrap samples, mean absolute error = 2.2%; Hosmer–Lemeshow test, *P* = 0.846). The 10-fold cross validation of the nomogram showed that the average misdiagnosis rate was 16.64%.

**Conclusion:**

The proposed model could be used to predict the probability of in-hospital death in patients undergoing CRRT for AKI after ATAAD surgery. It had the potential to assist doctors to identify the gravity of the situation and make the targeted therapeutic measures.

## Background

Acute type A aortic dissection (ATAAD) has a high incidence of postoperative acute kidney injury (AKI) due to its special pathophysiological changes and the surgical procedure, which seriously affects the patient's prognosis. AKI has a reported incidence ranging from 20 to 67%, according to the differences in the definition of AKI (1, 2). Some studies showed that the mortality due to postoperative AKI was 10–20 times higher than that without AKI in patients after ATAAD surgery (3, 4). In addition, the mortality for those in need of renal replacement therapy (RRT) was higher. The high risk of short-term mortality in patients undergoing RRT affects the prognosis of patients, making it necessary to identify prognostic factors and perform targeted interventions. Therefore, an effective model needed to be constructed for predicting the risk of in-hospital death in patients with AKI undergoing continuous renal replacement therapy (CRRT) after ATAAD surgery (in this study, all patients treated with RRT undergoing CRRT).

The nomogram has been considered as an effective way to create a straightforward visual graph of a numerical predictive model that quantifies the risk of a clinical outcome. This study aimed to identify the clinical risk factors for in-hospital death in patients with AKI undergoing CRRT after ATAAD surgery, and establish and validate a predictive model.

## Methods

### Patients

**Figure 1:**
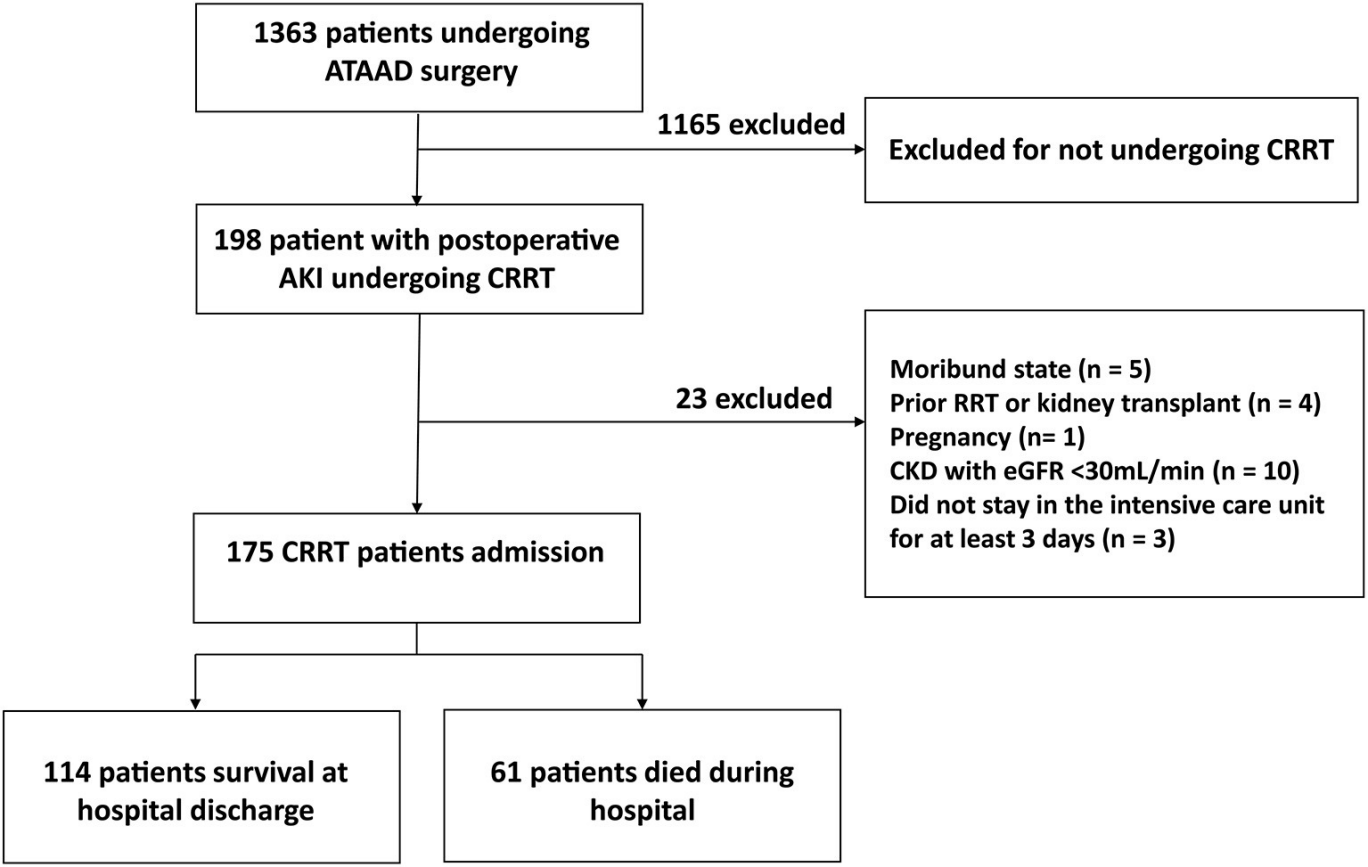
Study flowchart. OR, Odds ratio; CI, confidence interval; PND, permanent neurological dysfunction.

From June 1, 2015, to February 28, 2019, we retrospectively examined 175 patients with postoperative AKI who underwent ATAAD surgery and received CRRT in Beijing Anzhen Hospital, Capital Medical University (Figure 1). The inclusion criteria were as follows: (1) age ≥ 18 years, (2) undergoing ATAAD surgery with moderate hypothermia circulation arrest (MHCA) process, (3) CRRT due to postoperative AKI, (4) needing intensive care unit (ICU) treatment for at least 3 days. The exclusion criteria were as follows: (1) previous RRT or kidney transplant, (2) pregnant women, (3) moribund with expected death within 24 h, (4) patients who survived <12 h after CRRT, (5) previous chronic renal insufficiency (met any of the following criteria for more than 3 months: proteinuria ≥ 30 mg/24 h, urine albumin to creatinine ratio ≥ 3 mg/mmol, abnormal urine routine, electrolyte disorder caused by renal tubular damage, abnormal renal pathology, abnormal renal imaging, renal transplantation, and estimated glomerular filtration rate <30 ml/min).

AKI was diagnosed based on changes in the urine output, serum creatinine, or both, according to the Kidney Disease: Improving Global Outcomes (KDIGO) classification. Every patient had a urinary catheter to measure urine output every hour, and serum creatinine measurements were performed at least once daily.

### Data Collection

Relevant data related to the surgery were recorded. (1) The preoperative general data included age, gender, weight, height, time from the occurrence of dissection to the surgery, history of hypertension, type of dissection, maximum diameter of the aorta, cardiac function grade, myocardial ischemia, aortic regurgitation, renal insufficiency, pleural effusion, pericardial tamponade, aortic rupture, shock, smoking history, diabetes, oral administration of β-receptor blockers, and calcium antagonists. (2) The intraoperative data included operative time, cardiopulmonary bypass (CPB) time, circulatory arrest time, minimum temperature, crystal colloid input, and blood transfusion. (3) The postoperative data included blood pressure, central venous pressure, mechanical ventilation time, ICU stay, blood transfusion volume, and so forth. The complications included lung infection, respiratory failure, other organ dysfunction [Liver dysfunction is defined as serum alanine aminotransferase or aspartate aminotransferase that are at least 10 times the upper limit of normal value. Permanent neurological dysfunction (PND) is defined as a stroke due to embolism or hemorrhage, it is confirmed by consultation with a neurologist and by imaging (CT/MRI)], hemodynamic instability, arrhythmia, CRRT catheterization or anticoagulation-related bleeding, and electrolyte and acid–base imbalance. The urine volume per hour, daily intake and output, blood creatinine level, urea nitrogen, electrolyte, pH, and internal environment (whether acid–base balance) were recorded postoperatively. The patient treatment measures were recorded, including mechanical ventilation, vasoactive drug use, and fluid therapy.

The timing of most patients undergoing CRRT initiated within 8 h of AKI stage 3 using the KDIGO classification or if any of the following absolute indications for RRT were present: serum urea level > 40 mmol/L; Serum potassium concentration of >6 mmol/L despite medical treatment (bicarbonate and/or glucose-insulin infusion); pH <7.15 in a context of pure metabolic acidosis (PaCO2 below 35 mmHg) or in a context of mixed acidosis with PaCO2 ≥ 50 mmHg without the possibility of increasing alveolar ventilation.

Grouping: The patients were divided into survival and nonsurvival groups based on the vital status at hospital discharge.

### Statistical Analysis

Patients' baseline characteristics were expressed as frequency and percentage for categorical variables, and as mean ± standard deviation or median and interquartile range (IQR) for continuous variables, as appropriate. Indicators with missing values warranted interpolation by multiple imputations using the MICE package (5). We assumed that the data were missing at random (6); therefore, we performed predictive mean matching (7) to generate five complete imputed data sets that fit the logistic models. The binary data were tested using the χ^2^ test or Fisher exact test. Normally distributed data were compared for significance using *t*-tests. The Mann–Whitney *U*-test was applied for data with nonnormal distribution. The significance of each variable was assessed by univariate logistic regression analysis. The variables with *P-*value <0.1 were entered into the multivariate logistic regression analysis to identify the independent risk factors. Based on the results of the final regression analysis, a nomogram to predict the risk of in-hospital death in patients with postoperative AKI undergoing CRRT after ATAAD surgery was constructed using the R software (R software, version 4.1.2). The regression coefficients in multivariate logistic regression were proportionally transformed into a point scale, and the total points were converted into predicted probabilities (8).

The sample size calculation showed that a sample of 32 from the positive group and 60 from the negative group achieve 80% power to detect a difference of 0.15 between the area under the receiver operating characteristic (ROC) curve (AUC) under the null hypothesis of 0.85 and an AUC under the alternative hypothesis of 0.7000 using a two-sided z-test at a significance level of 0.05.

The performance of the nomogram was valuated by discrimination and calibration. The discrimination was demonstrated by the area under the ROC curve (equivalent to the C statistics). The calibration was performed using a visual calibration plot comparing the predicted and actual probabilities of in-hospital death. Furthermore, the calibration was performed using a visual calibration plot *via* 1,000 bootstrap resamples for internal validation to evaluate their predictive accuracies (9). The Hosmer–Lemeshow test was also recommended to assess calibration. Furthermore, we used 10-fold cross-validation to calculate the misdiagnosis rate. The statistical analysis and graphics were implemented by using R 4.1.2. All tests were two tailed, and a *P*-value <0.05 indicated a statistically significant difference.

## Results

A total of 175 patients with postoperative AKI undergoing CRRT after ATAAD were included in this study. The in-hospital death occurred in 61 (34.9%) patients. The comparison of the baseline data showed that the proportion of age > 65 years in the nonsurvival group was significantly higher than that in the survival group [age > 65 years: 18 cases (29.5%) in the nonsurvival group vs. 7 cases (6.1%) in the survival group, *P* <0.001]. Therefore, the age > 65 years was included in multiple logistic regression analyses. No significant differences were found in other baseline data between the two groups (Table 1).

**Table 1:** Baseline characteristics in the survival and nonsurvival groups.

**Variables**	**Survival group**	**Nonsurvival group**	***P***
Sex (male/female)	73/41	31/12	0.34
Age > 65 (%)	7 (6.1)	18 (29.5)	**<0.001**
BMI (kg/m^2^)	25.1 ± 3.5	25.7 ± 4.2	0.44
Hypertension (%)	82 (71.9)	49 (80.3)	0.22
CAD (%)	8 (7.0)	5 (8.2)	0.78
Diabetes (%)	9 (7.9)	6 (9.8)	0.66
Preoperative EF (%)	63.9 ± 6.1	61.0 ± 6.9	0.48
Creatinine (μmol/L, x ± S)	115.8 ± 84.2	117.9 ± 75.6	0.90
Urea nitrogen (mmol/L, x ± S)	8.9 ± 5.5	9.7 ± 5.9	0.55
Albumin (g/L, x ± S)	38.8 ± 7.7	36.9 ± 7.3	0.53
Myohemoglobin (μg/L, x ± S)	329.7 ± 197.6	285.7 ± 190.3	0.41
Leukocytes (10^9^/L, x ± S)	11.8 ± 4.4	12.6 ± 3.9	0.33
Hemoglobin (g/L, x ± S)	130.4 ± 20.6	131.4 ± 20.6	0.80

*BMI, Body Mass Index; CAD, Coronary atherosclerotic heart disease. Bold vaue indicates P < 0.05*.

The comparison of intraoperative data between the two groups revealed that the CPB time in the nonsurvival group was longer than that in the survival group (CPB time: nonsurvival group 235.6 ± 64.8 min vs. survival group: 219.6 ± 48.9 min, *P* = 0.04). Other intraoperative data, including the type of surgery, operative time, aortic cross-clamp time, MHCA time, and intraoperative blood transfusion volume, showed no statistical difference (Table 2). Therefore, CPB time was included in multivariate logistic regression analysis.

**Table 2:** Intraoperative variables in the survival and nonsurvival groups.

**Variables**	**Survival group**	**Nonsurvival group**	***P***
**Surgery type**			
Bentall replacement (%)	54 (47.4)	32 (52.5)	0.52
Total aortic arch replacement (%)	108 (94.7)	58 (95.1)	0.92
Partial aortic arch replacement (%)	6 (5.3)	3 (4.9)	0.89
Combined CABG (%)	10 (8.8)	1 (1.6)	0.07
Operative time (min, *x* ±*S*)	406.6 ± 185.6	442.6 ± 237.6	0.32
CPB time (min, *x* ±*S*)	219.6 ± 48.9	235.6 ± 64.8	**0.04**
Aortic cross-clamp time (min, *x* ±*S*)	120.6 ± 29.7	128.2 ± 37.6	0.11
MHCA time (min, *x* ±*S*)	22.8 ± 9.5	22.9 ± 9.2	0.97
Intraoperative infusion of RBC (u, Q1, Q3)	4.0 (2.0, 6.0)	4.0 (0.0, 10.0)	0.61
Intraoperative infusion of platelets (u, Q1, Q3)	0 (0, 0)	0 (0, 0)	0.87
Intraoperative infusion of plasma (u, Q1, Q3)	400.0 (0, 400.0)	400.0 (0, 800.0)	0.08

*CABG, Coronary artery bypass grafting; CPB, cardiopulmonary bypass; MHCA, moderate and hypothermia circulation arrest; RBC, red blood cell. Bold vaue indicates P < 0.05*.

The comparison of the clinical and laboratory data during CRRT between the two groups revealed that the lactic acid 6 h after CRRT, 12 h after CRRT, and 24 h after CRRT in the nonsurvival group was higher than that in the survival group (lactic acid 6 h after CRRT: 6.5 ± 4.9 mmol/L in the nonsurvival group vs. 3.7 ± 2.7 mmol/L in the survival group, *P* < 0.001; lactic acid 12 h after CRRT: 7.1 ± 5.2 mmol/L in the nonsurvival group vs. 3.1 ± 1.8 mmol/L in the survival group, *P* < 0.001; lactic acid 24 h after CRRT: 7.8 ± 5.9 mmol/L in the nonsurvival group vs. 2.7 ± 1.6 mmol/L in the survival group, *P* < 0.001). However, the OR of lactic acid 12 h after CRRT was the highest among them (lactic acid 6 h after CRRT: OR, 1.24; 95% CI, 1.10–1.40; lactic acid 12 h after CRRT: OR, 1.59; 95% CI, 1.34–1.89; lactic acid 24 h after CRRT: OR, 1.38; 95% CI, 1.18–1.60). Other CRRT data, including the duration of CRRT and laboratory indicators at the beginning of CRRT showed no statistical difference (Table 3). Therefore, lactic acid 12 h after CRRT was included in multivariate logistic regression analysis.

**Table 3:** Laboratory indicators during CRRT in the survival and nonsurvival groups.

**Variables**	**Survival group**	**Nonsurvival group**	***P***
Albumin upon initiation of CRRT (g/L, *x* ±*S*)	31.2 ± 13.2	28.8 ± 7.0	0.27
Leukocytes upon initiation of CRRT (G/L, *x* ±*S*)	24.1 ± 17.1	15.0 ± 6.9	0.28
BUN upon initiation of CRRT (ummol/L, *x* ±*S*)	20.9 ± 11.4	20.1 ± 12.3	0.67
Creatinine upon initiation of CRRT (mmol/L, *x* ±*S*)	289.9 ± 164.8	256.7 ± 123.3	0.23
Hemoglobin upon initiation of CRRT (g/L, *x* ±*S*)	94.1 ± 20.3	89.7 ± 18.5	0.11
Lactic acid upon initiation of CRRT (mmol/L, *x* ±*S*)	5.2 ± 4.7	6.4 ± 5.2	0.12
Lactic acid 6 h after CRRT (mmol/L, *x* ±*S*)	3.7 ± 2.7	6.5 ± 4.9	**<0.001**
Lactic acid 12 h after CRRT (mmol/L, *x* ±*S*)	3.1 ± 1.8	7.1 ± 5.2	**<0.001**
Lactic acid 24 h after CRRT (mmol/L, *x* ±*S*)	2.7 ± 1.6	7.8 ± 5.9	**<0.001**
Serum upon initiation of CRRT (mmol/L, *x* ±*S*)	5.6 ± 4.9	4.5 ± 2.7	0.32
Bicarbonate upon initiation of CRRT (mmol/L, *x* ±*S*)	24.0 ± 5.5	24.7 ± 4.2	0.50

*BUN, Blood urea nitrogen. Bold values indicates P <0.05*.

The comparison of postoperative complications and transfusion data during the ICU stay between the two groups: the proportion of liver dysfunction and PND in the nonsurvival group was significantly higher than those in the survival group [liver dysfunction: 24 cases in the nonsurvival group (39.3%) vs. 7 cases in the survival group (6.1%), *P* < 0.001; PND: 27 cases in the nonsurvival group (44.3%) vs. 12 cases in the survival group (10.5%), *P* < 0.001]. No significant differences were observed in other complications and the volume of blood transfusion during the ICU stay between the two groups (Table 4). Therefore, liver dysfunction and PND were included in multivariate logistic regression analysis.

**Table 4:** Postoperative complications and transfusion data during the ICU stay in the survival and nonsurvival groups.

**Variables**	**Survival group**	**Nonsurvival group**	***P***
Liver dysfunction (%)	7 (6.1)	24 (39.3)	**<0.001**
PND (%)	12 (10.5)	27 (44.3)	**<0.001**
Paraplegia inferior (%)	12 (10.5)	7 (11.5)	0.85
Catheter-related bloodstream infection (%)	2 (1.8)	3 (4.9)	0.23
Lung infection (%)	14 (12.3)	14 (23.0)	0.07
Gastrointestinal bleeding (%)	7 (6.1)	8 (13.1)	0.12
Infusion of RBC during the ICU stay (u, Q1, Q3)	12.0 (6.0, 18.0)	14.0 (5.5, 22.5)	0.52
Infusion of plasma during the ICU stay (ml, Q1, Q3)	400.0 (0, 600.0)	400.0 (0, 600.0)	0.99
Infusion of platelet during the ICU stay (u, Q1, Q3)	3.0 (1.0, 5.0)	2.5 (0.0, 4.0)	0.44

*PND, permanent neurological dysfunction. Bold values indicates P < 0.05*.

### Factors Selected for the Model

**Figure 2:**
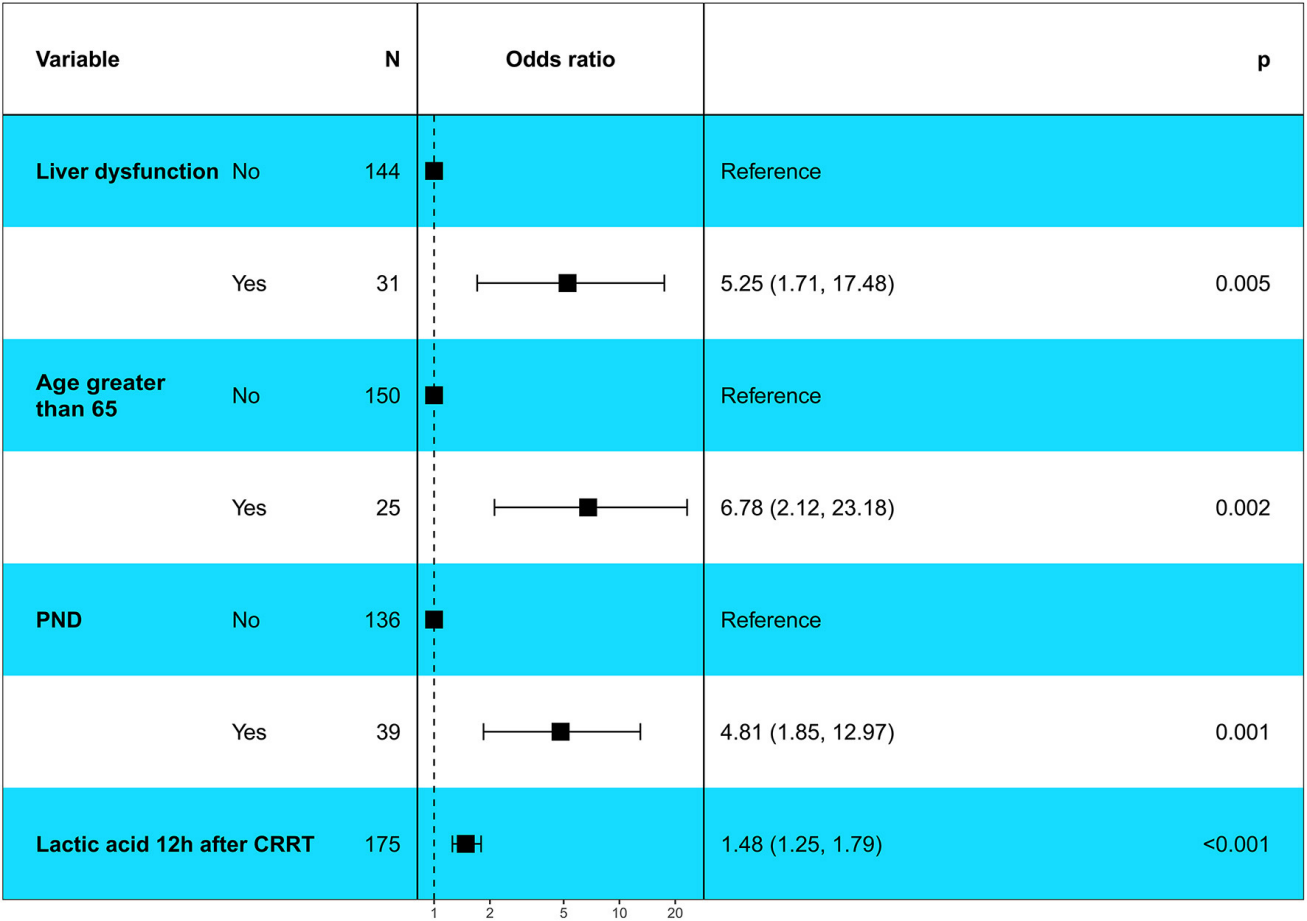
Multivariable logistic regression analysis showed the independent risk factors of in-hospital death in patients with postoperative AKI undergoing CRRT after ATAAD surgery. PND, Permanent neurological dysfunction.

Our study showed that the variables, including age > 65 years, lactic acid after 12 h of CRRT, liver dysfunction, PND and CPB time, had significant differences in the univariate logistic regression analysis (*P* < 0.1) and were included in multivariate logistic regression analysis. The result showed that age > 65 years (OR, 6.78; 95% CI, 2.12–23.18; *P* = 0.002), lactic acid 12 h after CRRT (OR, 1.48; 95% CI, 1.25–1.79; *P* < 0.001), liver dysfunction (OR, 5.25; 95% CI, 1.71–17.48; *P* = 0.005), and PND (OR, 4.81; 95% CI, 1.85–12.97; *P* = 0.001) were independent risk factors for in-hospital death in patients with AKI undergoing CRRT after ATAAD (Figure 2).

### Nomograms and Model Performance

**Figure 3:**
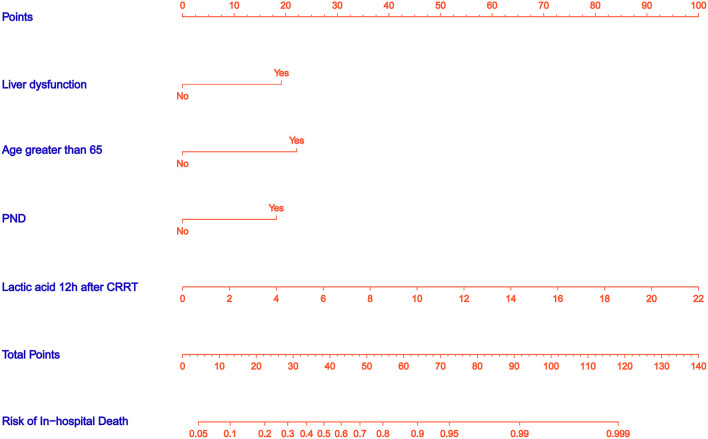
Nomogram predicts in-hospital death risk in patients with postoperative AKI undergoing CRRT after ATAAD surgery. The nomogram was established to predict the risk of in-hospital death based on four independent prognostic factors. The total score can be calculated by summation of single scores. We can estimate the probability of in-hospital death by projecting the total score to the lower total point scale.

**Figure 4:**
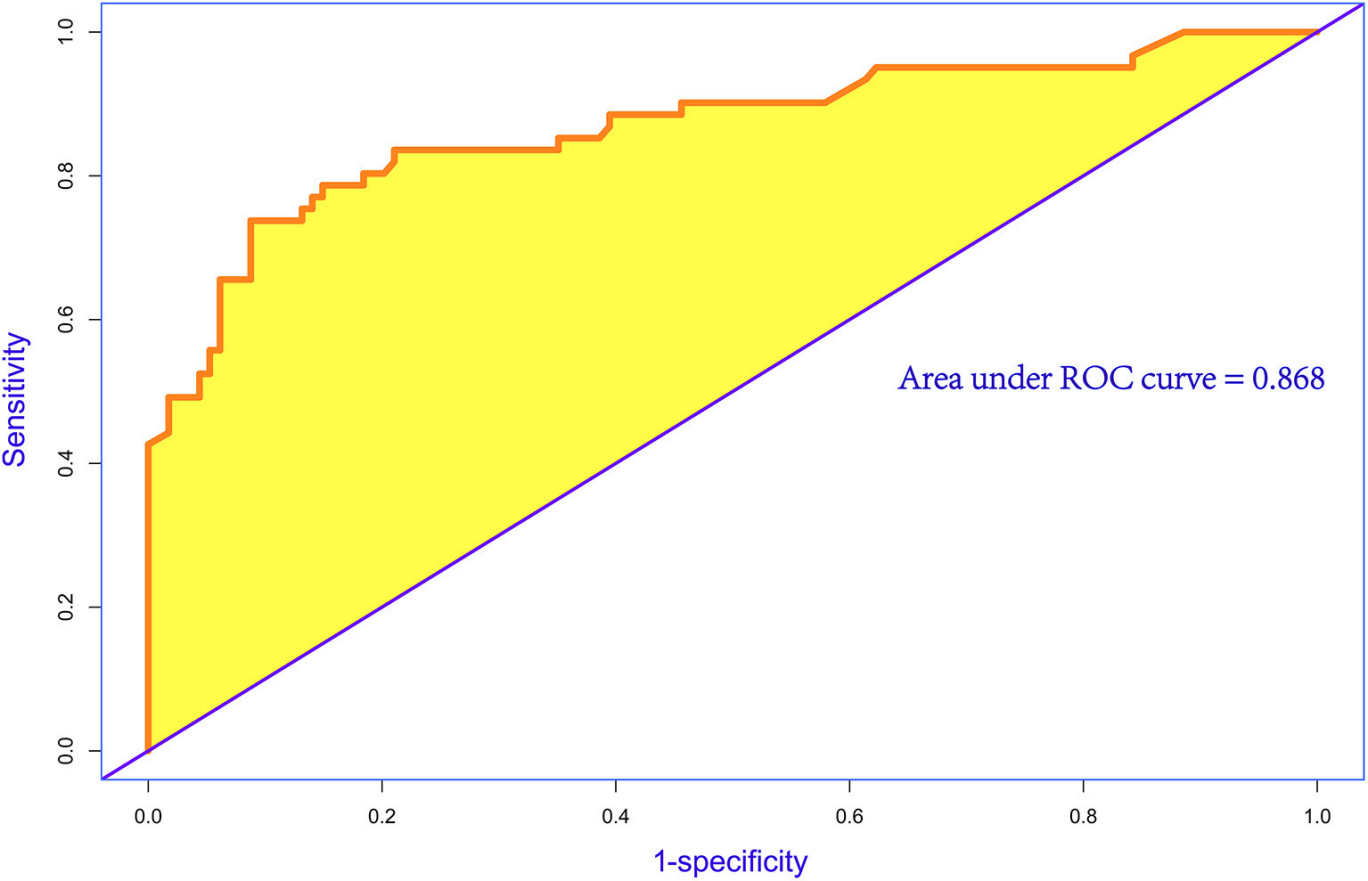
Receiver operating characteristic (ROC) curve for evaluating the discrimination performance of the model. The area under the ROC curve was 0.868 and C statistic was also 0.868.

**Figure 5:**
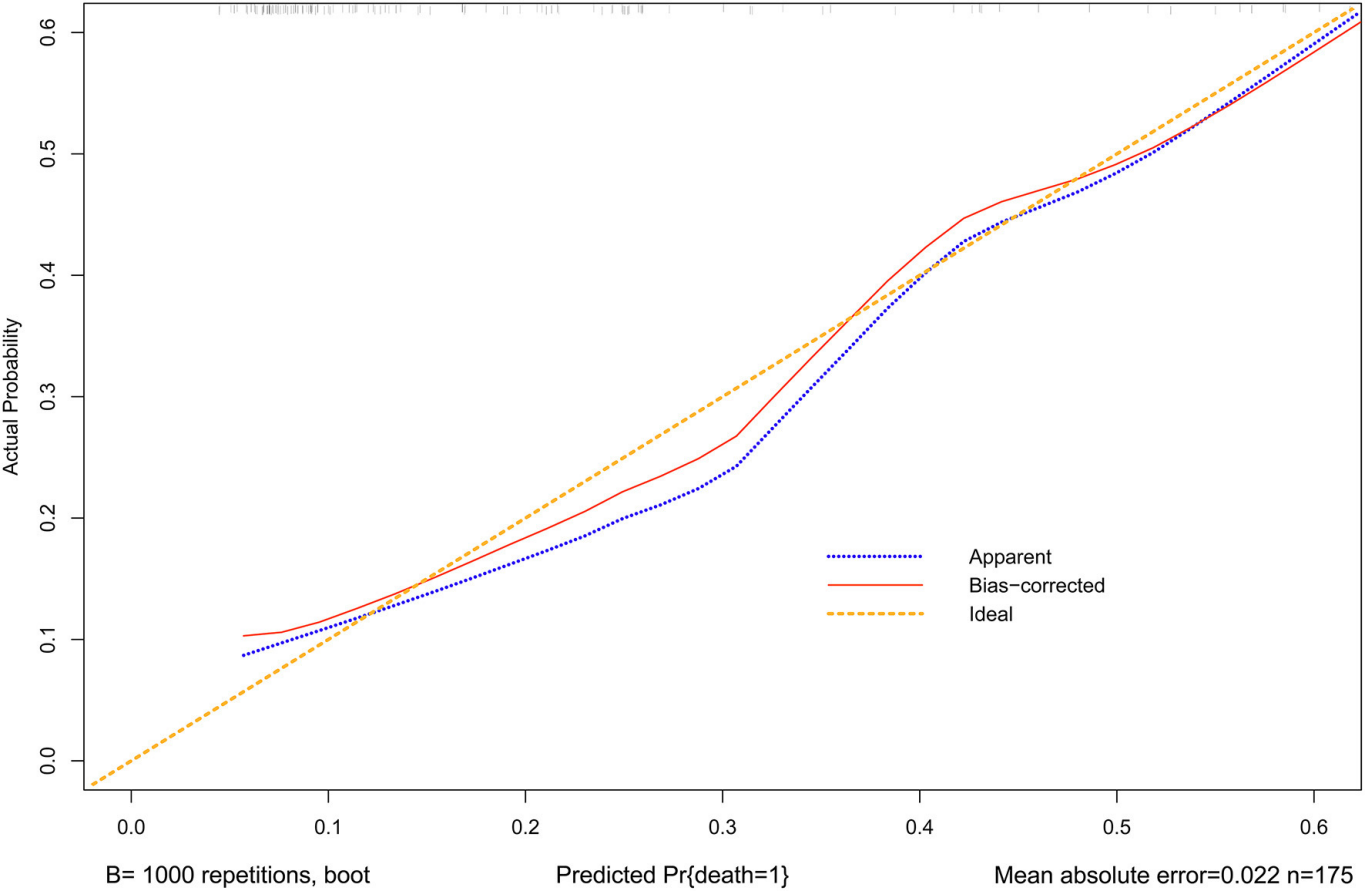
Calibration curves for the nomogram. The *x*-axis represents the nomogram-predicted probability, and the *y*-axis represents the actual probability of the nomogram. A perfect prediction would correspond to the 45° yellow dashed line. The blue dotted line represents the entire cohort (*n* = 175), and the red solid line is bias-corrected by bootstrapping (B = 1,000 repetitions), indicating observed nomogram performance. The mean absolute error = 0.022 (2.2%).

A nomogram was constructed to predict in-hospital death, including four significant independent risk factors: age > 65 years, lactic acid 12 h after CRRT, liver dysfunction, and PND (Figure 3). The total score was obtained by summing up the single scores used to estimate the probability of in-hospital mortality. The discrimination of the predictive model was estimated using a C statistic of 0.868 (95% CI, 0.806–0.930) and a bootstrap-corrected C statistic of 0.859; the area under the ROC curve was 0.868 (Figure 4). The calibration curve showed that the predicted probabilities of in-hospital death fitted well with the actual prevalence rates (calibration curve: *via* 1,000 bootstrap samples, mean absolute error = 0.022 (2.2%)) (Figure 5). The Hosmer–Lemeshow test (*P* = 0.846) also demonstrated good calibration. The 10-fold cross-validation of the nomogram showed that the average misdiagnosis rate was 16.64%.

## Discussion

Several factors have been reported to be associated with high mortality during CRRT (10–12). Researchers have been striving to develop prediction models for patients with AKI. However, These models have limited applicability to patients undergoing CRRT (13–15). For example, in a previous study, one model, HELENICC score, was suggested for patients undergoing CRRT (16). However, this study included only patients with septic AKI. Patients with postoperative AKI undergoing CRRT after ATAAD surgery that have higher in-hospital mortality and worse prognosis, making it necessary to screen for prognostic factors and perform targeted interventions. In this study, a nomogram was developed and validated for predicting the risk of in-hospital death in these patients. In additional, this nomogram had excellent discriminative performance and calibration, which provided individual predictions for each patient.

The present study created an uncomplicated intuitive graph of a statistical predictive model that quantified the risk of in-hospital death in patients with AKI undergoing CRRT after ATAAD surgery. In the proposed nomogram, age > 65 years was the greatest contributor to the risk of in-hospital death, followed by liver dysfunction and PND; lactic acid after 12 h of CRRT showed the smallest effect on the probability of in-hospital death.

This study showed that the in-hospital mortality rate was higher in patients aged more than 65 years and undergoing CRRT because of the decreased immune function of elderly patients, the physiological function of their organs degenerated, and the renal blood flow and glomerular filtration rate decreased every year with age, accompanied by hypertension, hyperlipidemia, diabetes, and other diseases. Therefore, they were more likely to have a poorer prognosis after CRRT for postoperative AKI. Commereuc and his colleagues (17) showed that the mortality of patients with AKI, who were older than 65 years and required CRRT in the ICU, was more than 70%, which was up to 76% in patients aged more than 80 years, with a significantly higher risk of death compared with patients aged <50 years. The prognosis of patients requiring CRRT was worse with increasing age. The aforementioned results also supported the conclusions of this study.

This study showed that high lactic acid values 12 h after CRRT was an independent prognostic factor for in-hospital death in patients undergoing CRRT for AKI after ATAAD. Blood lactic acid is an important indicator of systemic perfusion and oxygen metabolism; it reflects increased anaerobic metabolism in the presence of hypoperfusion (18). Elevated blood lactate levels have been shown to be a sensitive, early biochemical indicator of tissue hypoperfusion and oxygen insufficiency and can be used to assess disease severity and prognosis (19, 20). If patients do not get effective clearance of blood lactate in a short time, no improvement is seen in histiocyte hypoperfusion and oxygenation disorders, the progression of the disease worsens, shock and respiratory failure occur, and case fatality rate increases. If the clinical rescue treatment is appropriate, the tissue perfusion and oxygenation improve, the concentration of lactate in tissue cells decreases quickly, and the condition improves until recovery (21). Lactic acid remains high 12 h after CRRT, suggesting that the ischemic and hypoxic states of the tissues are still severe after CRRT, and the prognosis of such patients is poor.

In this study, liver dysfunction was a predictive factor for in-hospital death, which was defined as ischemic liver injury (ILI). A practical clinical definition of liver dysfunction is as follows: a syndrome with rapid and short-term increases in either AST or ALT levels to a level of more than 10 times the upper limit of normal, which is most usually occurred in critically ill patients. It is characterized by a predominant hepatocellular structure of damage, which is caused by insufficient blood and oxygen delivery to the liver cells. The latent etiologies often resulted in ILI are circulatory, cardiac or respiratory failure (22–24). Most specialists come to an agreement that outright conspicuous falls in systemic blood pressure are a typical predisposing characteristic of ILI. The incidence of ILI in the ICU (22, 25–28) was 1-12% and might be even higher in patients with cardiogenic shock (23, 25, 29). The surgical procedure for ATAAD is difficult, and the situation is full of challenges during the surgery. Malperfusion syndromes, such as liver dysfunction, can be present. If patients with AKI also have liver dysfunction, their in-hospital mortality increases significantly.

PND manifested mainly as stroke due to embolism or hemorrhage, which was diagnosed by a neurologist and confirmed by imaging (CT/MRI). Brain injury was one of the most important factors, other than cardiac insufficiency, leading to poor prognosis after cardiac surgery. Studies showed (30, 31) that the incidence of perioperative stroke was significantly higher in cardiac surgery than in noncardiac and non-neurosurgery. The incidence of perioperative stroke after cardiac surgery in patients with ATAAD was higher than that after other types of cardiac surgery (32). A deep hypothermic circulatory arrest (DHCA) has been shown to be one of the most risk factors for neurological complications after CPB. The incidence of stroke increased by 1.8–13.6%, and early mortality increased by 6.1–15% in adults after DHCA (33, 34). With the application of multiple brain protection strategies in patients with ATAAD, the incidence of neurological injury after ATAAD surgery is lower than before, but it is still an important factor affecting the prognosis of patients. The present study showed that patients requiring CRRT with PND had significantly increased in-hospital mortality. Multimodal brain function monitoring and the active use of multiple perioperative brain protection strategies during the perioperative period may improve patients' outcomes.

This study had a few limitations. First, although the internal validation of the model produced excellent discrimination and fabulous calibration, the generalizability of this nomogram still required external validation, especially from other countries, taking the differences in clinical behavior and epidemiology. Second, the prediction model was constructed retrospectively, and a retrospective research had its own limitations. It is necessary to carry out a prospective study to test the model. Third, the misdiagnosis rate of this model was still exists, and doctors used it should get noticed.

## Conclusions

In summary, a nomogram was developed and validated for predicting the risk of in-hospital death in patients with postoperative AKI undergoing CRRT after ATAAD surgery. The nomogram could help identify the gravity of the situation and provide treatment recommendations for these patients.

## Data Availability Statement

The original contributions presented in the study are included in the article/supplementary material, further inquiries can be directed to the corresponding author/s.

## Ethics Statement

This study was approved by the Institutional Ethics Committee of the Beijing Anzhen Hospital (No. KS2019034-3). All patients gave their written informed consent.

## Author Contributions

RJ carried out the studies, participated in collecting data, and drafted the manuscript. XL and ML participated in acquisition, analysis, or interpretation of data. NL, LS, and JZ reviewed and edited it. All authors contributed to the interpretation of the data and the completion of figures and tables and have read and approved the final manuscript.

## Funding

The study was supported by the Beijing Municipal Science and Technology Commission (No. Z191100006619095).

## Conflict of Interest

The authors declare that the research was conducted in the absence of any commercial or financial relationships that could be construed as a potential conflict of interest.

## Publisher's Note

All claims expressed in this article are solely those of the authors and do not necessarily represent those of their affiliated organizations, or those of the publisher, the editors and the reviewers. Any product that may be evaluated in this article, or claim that may be made by its manufacturer, is not guaranteed or endorsed by the publisher.

## Abbreviations

ATAAD: Acute Type A Aortic Dissection
AKI: Acute Kidney Injury
CRRT: Continuous Renal Replacement Therapy
BMI: Body Mass Index
CAD: Coronary Atherosclerotic Disease
CABG: Coronary Artery Bypass Grafting
CPB: Cardiopulmonary Bypass
MHCA: Moderate Hypothermic Circulatory Arrest
BUN: Blood Urea Nitrogen
RBC: Red Blood Cell
ICU: Intensive Care Unit
ILI: Ischemic Liver Injury
PND: Permanent Neurological Dysfunction.
